# Editorial: Interaction of nano and microplastic with different plant species: concerns and opportunities

**DOI:** 10.3389/fpls.2024.1378837

**Published:** 2024-02-16

**Authors:** Muhammad Arslan Ahmad, Muhammad Adeel, Muhammad Zain, Rabia Javed

**Affiliations:** ^1^ Shenzhen Key Laboratory of Marine Bioresource and Eco-environmental Science, College of Life Sciences and Oceanography, Shenzhen University, Shenzhen, China; ^2^ College of Physics and Optoelectronic Engineering, Shenzhen University, Shenzhen, China; ^3^ BNU-HKUST Laboratory of Green Innovation, Advanced Institute of Natural Sciences, Beijing Normal University at Zhuhai, Zhuhai, Guangdong, China; ^4^ Key Laboratory of Crop Genetics and Physiology of Jiangsu Province, Key Laboratory of Crop Cultivation and Physiology of Jiangsu Province, College of Agriculture, Yangzhou University, Yangzhou, China; ^5^ School of Science and the Environment, Memorial University of Newfoundland and Labrador, Corner Brook, NL, Canada

**Keywords:** microplastics, phytotoxicity, accumulation in soil, sustainable approaches, mechanistic understandings, food chain

Plastic pollution is a pervasive and growing problem. Plastic production is approximately 6300 million tons worldwide, of which 79% is deposited in landfills and other ecological segments leading to pollution (Geyer et al., 2017). The surface properties of nano-microplastics (NMPs) facilitate the adsorption of heavy metals, antibiotics, and other persistent organic pollutants, leading to their co-migration into the terrestrial environment (Guo et al., 2022). Plastic materials from various sources have been identified in agricultural soil, necessitating comprehensive investigations into the risks posed by NMPs and their consequences on agricultural plants (Azeem et al., 2021). Despite significant knowledge gaps in understanding the interactions between the natural environment and NMPs, mounting evidence suggests their detrimental effects on a diverse range of taxa (Azeem et al., 2022). It is imperative to explore the emerging hazards of NMPs in the soil-plant system and their potential effects on the food chain. The objective of this Research Topic is to elucidate key interactions (biochemical, cellular, and molecular) between different plant species and NMPs, focusing on relevant toxicity aspects. In addition, this Research Topic also focuses on the co-existence of NMPs with other environmental pollutants in soil-plant systems.

This Research Topic commences with an article by Luo et al. examining the impact of plastic mulching (PM) combined with nitrogen (N) fertilizer on winter wheat in dryland areas, focusing on grain yield, sulfur (S) nutrition, and quality. While PM enhances grain yield by 13.7%, it concurrently reduces grain S concentration, S requirement for 1,000 kg^–1^ grain, soil available S concentration, and post-anthesis S uptake. Shoot S accumulation at anthesis increases significantly under PM, though there is no notable difference at maturity. The relationship between grain S concentration/S requirement and N fertilization follows a linear-plateau pattern, peaking at 110/127 kg N ha^–1^ under PM, 37.5%/27.0% higher than under no mulching. The study suggests that combining soil S with optimal N fertilizer under PM enhances grain S concentration, benefiting nutritional and processing qualities in regions with similar cropping systems.

Lozano and Rilig explores the legacy effects of different types of MPs on plant-soil interactions. The research involved two phases; a conditioning phase where *Daucus carota* was grown with 12 types of MPs, and a feedback phase where soil inoculum from these conditioned soils was used to grow both native *D. carota* and the range-expanding plant species *Calamagrostis epigejos*. Results showed that films, foams, and fragments induced positive feedback on shoot mass, likely due to effects on soil water content and aeration during the conditioning phase. Films, by decreasing soil water content, may have reduced harmful soil biota, promoting mutualist lavishness, microbial activity, and carbon mineralization, clearly impacting plant growth. Foams and fragments, by enhancing soil aeration, likely increased mutualistic biota and enzymatic activity, supporting plant growth. However, fibers caused negative feedback on root mass, possibly by increasing soil water content and promoting soil pathogens. MPs also had a lasting impact on root traits, with *D. carota* developing thicker roots, likely to encourage mycorrhizal associations, and *C. epigejos* exhibiting reduced root diameter, possibly to minimize pathogenic infection. The legacy effects of MPs varied depending on the shape and polymer type, influencing plant-soil feedbacks. This research suggests that MPs can affect plant growth and soil interactions in diverse ways, and the legacy effects may contribute to the competitive success of certain plant species.

Han et al. investigates the impact of cadmium (Cd) alone and its co-existence with polypropylene microplastics (PP-MPs) on various aspects of wheat crop. The results indicate that while the germination rate of wheat was not considerably affected across treatment groups, the inhibitory effect on seed germination was intensified when Cd and PP-MPs co-existed. Germination index and mean germination time were not affected, but Cd and PP-MPs exhibited collective effects on germination energy. Cd alone and in combination with PP-MPs hindered wheat root and shoot length, with noticeable changes in seedling dry weight until the third day, followed by marginal differences on the seventh day. The combined application of Cd and PP-MPs showed that 50 µm (PP-MPs+Cd) had an hostile impact on wheat seedling growth, while 100 µm (PP-MPs+Cd) had a synergistic impact, likely due to the larger size of PP-MPs. The antioxidant enzyme system in wheat seeds and seedlings increased under single Cd pollution, but the activities of superoxide dismutase, catalase, and peroxidase decreased under combined pollution. Overall, the study suggests that Cd negatively affects wheat growth, and the co-existence of Cd and PP-MPs has complex effects influenced by the size of the PP-MPs.

Jia et al. examines the impact of microplastic (MP) pollution on plants in terrestrial ecosystems, highlighting its global significance. The study reveals that MP stress hampers plant’s physical growth through two main mechanisms; the blockage of openings in seed coats or roots, affecting water and nutrient uptake, and the induction of drought via enlarged soil cracking caused by MPs. Physiological growth reduction under MP stress is associated with excessive reactive oxygen species (ROS) production, altered leaf and root ionome, compromised hormonal regulation, and a drop in chlorophyll and photosynthesis. MP stress also ultimately affects plant growth by altering soil productivity, with the negative effects varying based on different MP surface functional groups and particle sizes. In response to these challenges, the review proposes agronomic tactics, including the use of growth regulators, biochar, substituting plastic mulch with crop residues, crop diversification, and biological deprivation, to mitigate the impact of MP stress on plants. However, the efficacy of these approaches is acknowledged to be MP-type-specific and dose-dependent.

Zhao et al. investigates the impact of graphene oxide (GO) on soybeans under drought stress, aiming to understand both physiological and molecular responses. GO-treated soybeans exhibited enhanced relative water content in stems and leaves by 127% and 128%, respectively, compared to wild-type (WT) plants. Root parameters in GO-treated soybeans, including total root length, surface area, diameter, and volume, improved by 33%, 38%, 34%, and 35% compared to control. Enzyme activities related to plant defense mechanisms, such as superoxide dismutase, catalase, peroxidase, and ascorbate peroxidase, increased by 29%, 57%, 28%, and 66%, respectively, while indicators of oxidative stress (relative conductivity, malondialdehyde, and hydrogen peroxide) decreased significantly. Drought-related hormones in GO-treated soybeans increased by 32%, 34%, and 67% compared to control. Overall, the study suggests that GO treatment enhances soybean defense mechanisms against drought, providing potential prospects for improving drought tolerance through operative soil water retention agents.

Verma et al. underscores the pervasive environmental impact of plastics, particularly microplastics (MPs) and nanoplastics (NPs), on agro-ecosystems and plant development. The extensive use and inadequate recycling of plastics lead to the widespread dispersal of plastic waste in the atmosphere and land. The contamination of the environment and farmland soils with MPs and NPs negatively affects agro-ecosystem functioning. The study emphasizes the potential contributions of recyclable organic waste, plastic mulching, and plastic deposition in agroecosystems, highlighting the need to understand their hazardous impacts. Notably, the review draws attention to the dissolution of bioplastics into MNPs, an aspect often overlooked in recent studies focusing on agro-ecosystems. The distribution, concentration, fate, and main sources of MNPs in agroecosystems needs comprehensive understanding. While acknowledging the limited findings, review emphasizes on the importance of addressing knowledge gaps to safeguard global food safety and security in the face of micro and nanoplastic pollution in farming systems.

Overall, the papers contained within this Research Topic introduce and discourse the impacts of NMPs on soil plant systems. There is still a long way to completely understand and avoid the growing concerns of plastic pollutions and offer creative pathways for sustainable environmental and agricultural practices. Far-reaching interdisciplinary research and innovative methodological advancements are likely to pave the way toward zero plastic pollution. In short, some of the research problems have been addressed by the research published in this Research Topic while few still need attentions.

## Author contributions

MAA: Conceptualization, Project administration, Writing – original draft, Writing – review & editing. MA: Writing – original draft, Writing – review & editing. MZ: Writing – original draft, Writing – review & editing. RJ: Writing – original draft, Writing – review & editing.
